# Do water-limiting conditions predispose Norway spruce to bark beetle attack?

**DOI:** 10.1111/nph.13166

**Published:** 2014-11-21

**Authors:** Sigrid Netherer, Bradley Matthews, Klaus Katzensteiner, Emma Blackwell, Patrick Henschke, Peter Hietz, Josef Pennerstorfer, Sabine Rosner, Silvia Kikuta, Helmut Schume, Axel Schopf

**Affiliations:** 1 Institute of Forest Entomology, Forest Pathology and Forest Protection, Department of Forest and Soil Sciences, BOKU – University of Natural Resources and Life SciencesHasenauerstr. 38, A-1190, Vienna, Austria; 2 Institute of Forest Ecology, Department of Forest and Soil Sciences, BOKU – University of Natural Resources and Life SciencesPeter-Jordan-Str 82, A-1190, Vienna, Austria; 3 Institute of Botany, Department of Integrative Biology and Biodiversity Research, BOKU – University of Natural Resources and Life SciencesGregor-Mendel-Str 33, A-1190, Vienna, Austria

**Keywords:** climate change, defence capability, drought stress, host acceptance, *Ips typographus*, Norway spruce (*Picea abies*), predisposition, tree physiology

## Abstract

- Drought is considered to enhance susceptibility of Norway spruce (*Picea abies*) to infestations by the Eurasian spruce bark beetle (*Ips typographus*, Coleoptera: Curculionidae), although empirical evidence is scarce. We studied the impact of experimentally induced drought on tree water status and constitutive resin flow, and how physiological stress affects host acceptance and resistance.
- We established rain-out shelters to induce both severe (two full-cover plots) and moderate (two semi-cover plots) drought stress. In total, 18 sample trees, which were divided equally between the above treatment plots and two control plots, were investigated. Infestation was controlled experimentally using a novel ‘attack box’ method.
- Treatments influenced the ratios of successful and defended attacks, but predisposition of trees to infestation appeared to be mainly driven by variations in stress status of the individual trees over time. With increasingly negative twig water potentials and decreasing resin exudation, the defence capability of the spruce trees decreased.
- We provide empirical evidence that water-limiting conditions impair Norway spruce resistance to bark beetle attack. Yet, at the same time our data point to reduced host acceptance by*I. typographus* with more extreme drought stress, indicated by strongly negative pre-dawn twig water potentials.

## Introduction

Drought has been identified as one of the main triggers of bark beetle (Coleoptera: Curculionidae, Scolytinae) outbreaks in conifer stands (Rouault*et al*., 2006; Dobbertin*et al*., 2007; Seidl*et al*., 2011; Kaiser*et al*., 2013; Williams*et al*., 2013). Infestation of Norway spruce (*Picea abies*) forests by the Eurasian spruce bark beetle (*Ips typographus*), a major biotic forest disturbance agent in Europe, was found to be significantly correlated with summer precipitation deficits and increased temperature (Baier*et al*., 2007; Faccoli, 2009; Marini*et al*., 2012, 2013). Environmental stress significantly impacts host colonization and may provoke transitions from endemic to epidemic bark beetle development (Berryman*et al*., 1989; Raffa*et al*., 2008; Kausrud*et al*., 2012). Various studies have demonstrated the relationship between bark beetle attack and pre-dawn leaf/twig water potential, a proxy for drought stress status (Sellin, 1997). Breshears*et al*. (2009) found pinyon pine (*Pinus edulis*) trees with pre-dawn leaf water potentials substantially below their zero carbon assimilation point being associated with*Ips confusus* infestation and tree dieback. A meta-study by Jactel*et al*. (2012) revealed a positive correlation between the damage level on woody organs caused by secondary agents and the ratio between pre-dawn leaf water potential and water potential inducing 50% loss in hydraulic conductivity. However, physiological stress proxies or threshold values indicating increased susceptibility have yet to be published for Norway spruce. This study is the first to investigate how drought stress of mature Norway spruce trees influences both their acceptance as a host by*I. typographus* and their ability to defend against this pest.

When bark beetles attack conifer tree species, the periderm (Rosner & Führer, 2002) and the constitutive defence system are the first barriers they must overcome. The constitutive defence system is built from resin ducts, resin blisters and resin cells (Franceschi*et al*., 2005). Epithelial cells arranged around the resin ducts synthesize terpenoid resins, which perform wound cleansing and sealing, and prevent invasion of biotic agents (Lieutier, 2004). Phloem and bark thickness as well as resin duct density have been found to be important anatomical features influencing resistance of Norway spruce against borings by*I. tyopgraphus* (Baier, 1996). Resistance to bark beetles was furthermore found to be strongly dependent on the intensity of resin exudation (Lorio & Hodges, 1968; Lieutier, 2004; Boone*et al*., 2011). Although defence capacity varies from tree to tree as a result of*inter alia* genetic variation and age, inherent resistance to bark beetles is thought to be compromised by environmental stresses, such as drought. During periods of reduced rainfall trees can become drought-stressed when water loss via transpiration exceeds uptake from the soil, causing tree water deficits (Bréda*et al*., 2006; Gartner*et al*., 2009). Severe stress can develop when late afternoon and evening uptake from the soil cannot replenish the water previously transpired (Kozlowski, 1979; Kozlowski*et al*., 1991). As drought persists, water potential and hydraulic conductivity in the soil and thus in the xylem conduits decrease, which increases the risk of cavitation and embolism in the conducting tissues (Tyree & Sperry, 1989; Cruiziat*et al*., 2002; Cochard*et al*., 2009). Species such as Norway spruce avoid/delay the decrease in xylem water potentials by restricting transpiration via stomatal closure (Rothe*et al*., 2002; Schume*et al*., 2004). While this strategy can help maintain xylem water potentials above critical thresholds, the reduced ascent of water and leaf–atmosphere gas exchange cause reductions in photosynthetic production despite continued respiratory consumption of carbon (Bréda*et al*., 2006; Rennenberg*et al*., 2006). Stored carbon reserves potentially become depleted (McDowell*et al*., 2008), significantly reducing the carbohydrates available for synthesis of defence structures and compounds under persistent drought. Indeed, it has been recently suggested that this mechanism of ‘carbon starvation’ may be partially driving the correlation between drought occurrence and insect infestations (Allen*et al*., 2010; McDowell*et al*., 2011).

While enhanced bark beetle attack and tree mortality have been demonstrated in pine stands where drought has been artificially induced (Lorio*et al*., 1995; Saracino*et al*., 1997; Gaylord*et al*., 2013), the relationship between drought stress and successful insect attack is far from linear (Lieutier, 2004). In the case of*Tomicus destruens*, shoots of vigorous trees rather than weakened ones were preferred during maturation feeding (Branco*et al*., 2010), which is a common observation among chewing insects (Koricheva*et al*., 1998). The intensity, duration and continuity of drought regimes strongly influence tree–herbivore interactions (Huberty & Denno, 2004). Moderate drought is hypothesized to limit tree growth but to enhance carbon allocation to constitutive defences, while severe drought should constrain the production of secondary metabolites such as oleoresin as a result of the depletion of carbon reserves (Herms & Mattson, 1992; Ayres, 1993; Christiansen & Bakke, 1997; Lombardero*et al*., 2000; Desprez-Loustau*et al*., 2006). Nevertheless, high levels of tree resistance to bark beetles do not necessarily result in low attack and vice versa. Successful host approach, entry and brood establishment are subject to different internal thresholds and control processes ranging from insect physiological state and population density, to chemical stimuli from aggregation pheromones and/or host tissues (e.g. concentrations and emission of monoterpenes) (Raffa*et al*., 2008). The intensity of resin flow (RF) and monoterpene: pheromone ratios were, for instance, shown to influence bark beetle aggregation in a dose-dependent manner (Erbilgin*et al*., 2003). Investigating drought effects on host susceptibility is thus complicated by myriad internal and external factors influencing such tree–herbivore relationships.

To uncover some of the complex mechanisms described above, in 2011 we initiated the Rosalia Roof Project, the first*in situ* manipulation experiment investigating how drought stress affects the vulnerability of Norway spruce to infestation by*I. typographus*. We manipulated soil moisture by establishing rain-out shelters and noncovered control plots in a mature Norway spruce stand and measured a number of tree physiological parameters. To evaluate the incidence and success of bark beetle infestation on our sample trees, a novel experimental approach allowed us to control the timing and intensity of the beetle pressure and, moreover, offered the beetles a choice between attacking the trees or not. This study addresses the following research questions: How does the physiological state of the sample trees, indicated by the pre-dawn twig water potential, osmotic potential of the secondary phloem and RF, vary seasonally? Is attack activity of*I. typographus* subject to seasonal changes and are ratios of successful and defended attacks related to the underlying drought treatment? Which tree physiological variables most significantly explain successful and defended attacks?

## Materials and Methods

### Study site

Our study site was located in the Rosalia Mountains, Lower Austria (47.689676N, 16.294084E; decimal degrees, WGS84), in the eastern foothills of the Alps. The experimental plots were set up in a 90-yr-old Norway spruce (*Picea abies* L.) stand interspersed with European beech (*Fagus sylvatica*) on a moderately northwest exposed slope at 700 m above sea level (asl). Mean annual temperature at a nearby weather station (1990–1999) is 6.5°C, with mean annual precipitation (1990–1999) being 796 mm. In this region,*c*. 60% of precipitation falls from May to September. Winter precipitation is low, and snow cover commonly persists for no longer than 5–6 wk (Gasch, 1985). According to the World Reference Base (WRB) classification system (IUSS Working Group WRB, 2006) the soil at the study site is a Haplic Cambisol (Dystric, Endoskeletic) developed from colluvial debris of gneiss and micashist.

### Experimental set-up

**Figure 1:**
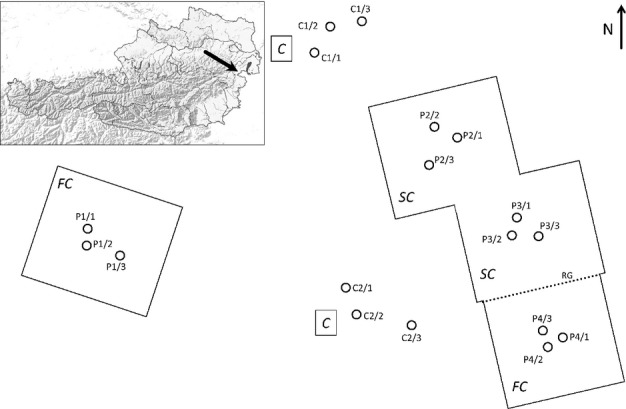
Layout of the experimental site located in the Rosalia Mountains, Lower Austria, established on a northwest exposed, slightly inclined upper slope; the set-up includes two different drought treatments each enclosing three Norway spruce (*Picea abies*) sample trees (FC, full-cover plots, 18.0 × 20.0 m including P1/1–P1/3; 18.0 × 18.0 m including P4/1–P4/3; SC, semicover plots, each 18.0 × 20.0 m; P2/1– P2/3, P3/1–P3/3) and two controls without roofs (C, control plots; C1/1–C1/3, C2/1–C2/3). At the lower edge of the FC plot directly adjacent to the SC plots, a rain gutter (RG) was installed in order to drain water off the roof.

Drought (full and partial) was induced in spring and summer of 2012 and 2013 by constructing rain-out shelters beneath the canopy of the study site at*c*. 1.20 m stem height. Fluted polyester roofs strengthened by fibreglass (light-permeable and UV-resistant) were established on a wooden construction built over a total area of 1404 m^2^ in June 2012. Gaps around the trees enclosed by the roofs were sealed with pond liner. The experimental set-up comprised full cover (FC), semi-cover (SC; 50% closure by partial cover) and control (C) treatments; each with two replicates (Fig.1). Three Norway spruce trees in the centre of each plot were selected as sample trees for monitoring tree physiological/defence parameters, and for attack experiments. To ensure similar initial physiological conditions across treatments, plots were only chosen where the respective sample trees contributed to the upper canopy and exhibited healthy crowns. In January 2012, one SC sample tree was thrown over by a storm and replaced by an adjacent spruce tree. Autumn and spring precipitation was simulated on the FC plots by sprinkling with ground water provided by the local fire service. Each FC plot was irrigated twice before winter (6–7 and 13–14 November 2012), and twice afterwards (24 April and 7–8 May 2013), with both plots receiving 178 mm water in total. For all treatments, 30-cm and 60-cm wave guide pairs were installed in the soil in a systematic spatial design in April 2012. Soil volumetric water content (SWC) over 0–30 cm and 0–60 cm soil depth was measured discontinuously by connecting to the wave guides with a portable Time Domain Reflectometry (TDR) measurement unit (Trase system 1; Soilmoisture Equipment Corp., Santa Barbara, CA, USA). Air temperature, relative humidity, wind speed, global radiation and precipitation were logged every 15 min by a weather station installed in a nearby clearing.

### Tree physiological indicators of drought stress and resistance to*I. typographus*

Tree physiological parameters were measured on all 18 sample trees during the growing seasons of 2012 and 2013 (Table1). Pre-dawn twig water potential (Ψ_t_) was measured with a pressure chamber (Soilmoisture Equipment Corp.) using twigs from the upper half/third of the tree crowns. Sampling always began 1.5 h before sunrise. Twigs were sampled by climbing one tree per treatment plot on 19 June 2012, and were obtained from all sample trees using a shotgun on all subsequent dates. The length of the current year's shoots and needles was recorded per study tree on four dates (27 June, 24 July, 21 August, and 25 September 2013).

**Table 1:** Summary of parameters measured during the study detailing the respective measurement/sampling dates (day-month) and abbreviations (Abbr) in the text; tree physiological parameters and bark beetle (*Ips typographus*) attacks are recorded on Norway spruce (*Picea abies*) sample trees

Parameter	Abbreviation	Measurement/sampling date	
		2012	2013
Volumetric soil water content over 0–30 and 0–60 cm depth	SWC	30-04, 17-05, 01-06, 16-06, 04-07, 18-07, 31-07, 15-08, 07-09, 28-09	23-04, 08-05, 21-05, 29-06, 09-07, 23-07, 07-08, 20-08, 24-09
Pre-dawn twig water potential	Ψ_t_	19-06, 08-08, 20-08, 05-09, 20-09	22-05, 27-06, 24-07, 25-09
Relative water content of the xylem and secondary phloem	RWC	14-05, 19-06, 27-08	27-06, 23-07, 21-08, 25-09
Osmotic potential of the secondary phloem at full saturation	Ψ_o_	14-05, 19-06, 27-08	27-06, 23-07, 21-08, 25-09
Constitutive resin flow on the stem	RF	15-05, 20-06, 22-08, 02-10	22-05, 27-06, 10-07, 24-07, 22-08, 25-09
Bark anatomy	n.a.		22-05, 22-08
Bark beetle attack with attack boxes	n.a.		21/22-05, 05/06-06, 18/19-06, 26/27-06, 02/03-07, 09/10-07, 16/17-07, 23/24-07, 06/07-08, 20/21-08, 24/25-09

n.a., not applicable.

Relative water content (RWC) was measured for sapwood and secondary phloem (hereafter termed ‘phloem’), and osmotic potential at full saturation (Ψ_o_) was measured for phloem only. Bark and outer sapwood were sampled at breast height with a cork borer (6 mm diameter) at noon (Table1). Samples comprising the bark and one to four annual rings of the outer sapwood were stored and transported as described in Rosner*et al*. (2001). Bark was separated from sapwood with a sharp razor blade. The whole sapwood sample and the conducting phloem disc (i.e. a thin slice of 0.2–0.5 mm next to the cambium) cut from the bark were placed on a micro-balance with a resolution of 1 μg (MT5; Mettler, Zürich, Switzerland). After determining the fresh weight (FW), the phloem discs were saturated with ultra-pure water (milli-Q; Merck KGaA, Darmstadt, Germany) on polyurethane foam in a small plastic box (Rosner & Kikuta, 2002). The discs were placed in small holes in the foam with the same diameter as the samples, providing direct contact between the samples and the foam. After measuring saturated weight (SW), the phloem discs were stored at −20°C. Sapwood samples were saturated in ultra-pure water under a partial vacuum (Hietz*et al*., 2008). Subsequent to determination of SW (for phloem after determining Ψ_o_) the discs were dried to constant weight for 48 h at 90°C and weighed (DW). The RWC of phloem and sapwood was calculated as:


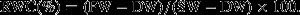
Eqn 1

We measured Ψ_o_ at full saturation for the phloem discs following the technique described by Rosner & Kikuta (2002). The frozen discs were cooled down to −80°C for 1 d in order to eliminate turgor potential, quickly thawed at ambient temperature inside the vials, and placed into the sample holder of the Wescor 5500 Vapor Pressure Osmometer (Wescor Inc., Logan, UT, USA).

RF at breast height of sample trees was recorded for c. 12 h (19:00–07:00 h) on opposite stem sides by inserting glass tubes with 3 mm inner diameter and 103 mm length into holes made by a cork borer. We found no significant differences between the two replicates per sample tree in 2012 (data not shown) and therefore used mean values of the two replicates for further analysis. We calculated resin flow rate as mm^3^ h^−1^ exuded resin on four dates in 2012 and six dates in 2013 (Table1). In May and August 2013, extra cores were immediately sealed in tubes, cooled and after transport conserved at −20°C for the analysis of bark anatomy. The frozen phloem samples were put in tissue embedding medium (GSV1; SLEE Medical GmbH, Mainz, Germany) and 25-μm sections from the phloem tissue next to the cambial zone were produced in a cryotome (SLEE Medical) at a cabinet temperature of −20°C. Analysis was performed using a transmitted light/bright-field microscope (Axiophot; Zeiss) with a high-resolution camera (AxioCam MRc5(D); Zeiss) connected to a computer, on which axiovision Release 4.7.1 (Carl Zeiss GmbH, Vienna, Austria) was installed. We counted the total number of resin canals and the number of surrounding epithelial cells per cm^2^ and measured resin canal area using datinf measure Version 2.1 (datinf GmbH, Tübingen, Germany).

### Rearing of*I. typographus*

Eurasian spruce bark beetles,*Ips typographus* L. (Coleoptera; Curculionidae), were reared for the attack experiments in the laboratory (photoperiod, 16 h : 8 h, light : dark; 25°C constant temperature) on spruce bolts. Thus, a sufficient number of beetles of comparable life history and maturity were available any time during the experimental season. For each attack experiment, we collected beetles that had recently emerged from the galleries and transferred them to the field site in a cooled box. Proportions of males and females were assumed to be equal, but were checked by dissection after the experiments.

### Monitoring of bark beetle attack

We designed ‘attack boxes’ for the purpose of controlled experimental testing of*I. typographus* attacks. The boxes (43 × 23 × 25 cm) were constructed from spruce plywood, with the outer wall made of transparent acrylic glass and the tree-facing side left open to be fitted against the stem at 5–6 m stem height (Supporting Information Fig. S1). A small plastic container for the collection of exiting beetles was attached to the outer wall. Attack experiments were performed 11 times from the end of May to the end of September 2013 (Table1) and started around noon. The ‘attack period’ for each tree began once a plastic bottle containing 20 beetles and some paper tissue had been connected to the box. Box temperatures were repeatedly monitored using temperature sensor-data logger systems (Tinytag Plus; Gemini Dataloggers Ltd, Chichester, UK) and closely followed forest air temperatures (data not shown). After 24–26 h of exposure, the boxes were removed; the numbers of beetles still present in the initial bottle, inside the box and in the exit container were recorded. The exposed bark area was thoroughly examined for fresh boring holes. Failed attempts of the beetles to enter the bark that provoked RF were counted as ‘defended’ by the tree, regardless of whether the beetles died in the exuded resin or exited the entry site. Boring holes in combination with dry boring dust and/or no RF were recorded as ‘successful’ infestation. Beetles were recollected, frozen at −20°C and later sexed under a light microscope. We pooled the numbers of defended and successful attacks into four periods of similar weather conditions and attack intensities: (1) spring (21–22 May, 5–6 June and 18–19 June; but not 26–27 June because of inactivity of beetles as a consequence of temperatures below 15°C), characterized by high rainfall, low to moderate temperature, and high numbers of attacks; (2) early summer (2–3 July, 9–10 July and 16–17 July), with little rainfall, moderate temperatures, but minor attack activities; (3) mid-summer (23–24 July and 6–7 August), with little rain, high temperatures and intense infestation; (4) late summer and autumn (21–22 August and 24–25 September), when precipitation increased, temperatures decreased, and attack was seldom.

### Statistical analyses

Effects of treatment (independent variable) and recording date (repeated factor) on SWC, RF, RWC of phloem and sapwood, Ψ_o_, Ψ_t_, and shoot and needle lengths were analysed by separate repeated measures MANCOVA. One-way ANOVA (level of significance*P* < 0.05) was used to evaluate each date individually in the case of a significant interaction of treatment and date, and to test for differences in attack by period. Post hoc comparisons of means for treatments and dates were made using the Scheffé test.

We performed linear regression analyses to evaluate the influence of air temperature on RF and the relationships between Ψ_t_ and RF. Based on the relationship between air temperature and RF, temperature-standardized values of RF at 15°C (RF_15_) were calculated for each treatment and each sampling date by multiplying the slope by the mean temperature of the sampling date minus 15°C, and subtracting this figure from the treatment mean RF. We further investigated the influence of maximum temperature during the attack experiments on the proportions of beetles leaving the initial bottle and entering the exit container. Pearson's correlation test (two-tailed) was used to investigate relationships between the various measured anatomical, physiological, and beetle attack parameters.

We conducted χ^2^ analyses with a null hypothesis of an even distribution of successful and defended attacks by*I. typographus* to investigate the impact of treatment over the whole season and during each of the four periods. For each period, we modelled the influence of tree physiological parameters on successful and defended attack ratios by Poisson log-linear regression (generalized linear model (GLM)).

To investigate the distribution and movement of the sample trees between different states of drought stress and defence capability over the 2013 study period, a principal components analysis (PCA) was performed. The PCA was conducted with transformed values of RF and Ψ_t_, and the mean air temperature and maximum vapour pressure deficit recorded the day before the sampling date. Consequently, each tree for each sampling date was treated as an individual observation. The above parameters were transformed by taking the square root of the value (in the case of RF, the square root of (RF + 1)), so as to satisfy the PCA criterion of normally distributed variables, confirmed by the Kolmogorow–Smirnov test (*P* < 0.05). The suitability of the matrix for PCA was checked by the Kaiser–Meyer–Olkin (KMO) measure of sampling adequacy (KMO 0.755). To simplify interpretation, the first two principal components were retained from the PCA and rotated (varimax rotation), thus minimizing the number of variables loading on each component, while maximizing the respective component loadings. The component scores of the observations (i.e. each sample tree per date) were calculated by the regression method. Statistical analyses were performed in ibm spss statistics (ver. 19; IBM Corp., Armonk, NY, USA) and R (R Core Team, 2014).

## Results

### Weather and soil water conditions

**Figure 2:**
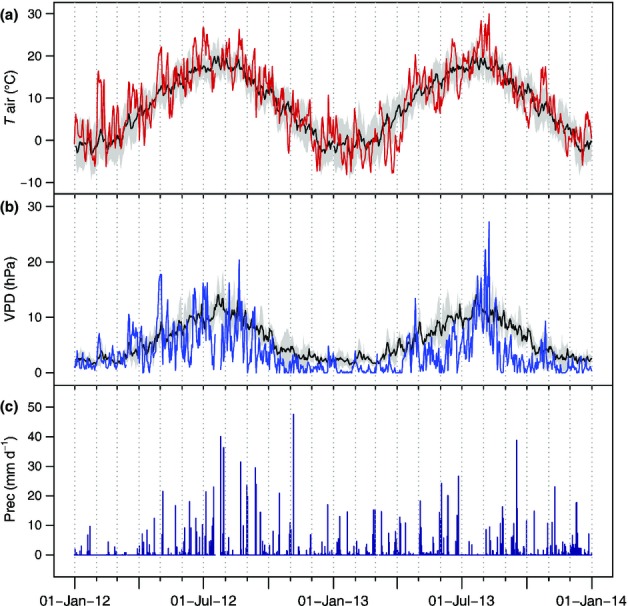
Climatic conditions recorded at a climate station located in a clearing 200 m from the experimental site, from 1 January 2012 to 1 January 2014: (a) daily mean air temperature (*T*_air_), (b) daily mean vapour pressure deficit (VPD), and (c) daily sum of precipitation (Prec). Values from the site weather station are plotted in colour, while mean values of daily temperature and VPD averaged over 2001–2011 from a nearby climate station (Heuberg; 640 m above sea level) are plotted beneath in black with the grey area delineating ± 1 SD.

Annual mean daily air temperature and annual precipitation sum were similar in the two years of the study (8.95°C and 742 mm in 2012, and 8.26°C and 774 mm in 2013), although 2013 exhibited greater variation from average temporal trends observed in the area (Fig.2). During July and August, conditions were substantially hotter and drier in 2013 than in 2012 (87 mm precipitation and 8 d where mean daily air temperature exceeded 25°C in 2013 compared with 254 mm and 2 d > 25°C in 2012). Correspondingly, daily mean vapour pressure deficit reached a maximum value of 27.23 hPa in 2013 and 20.35 hPa in 2012.

**Figure 3:**
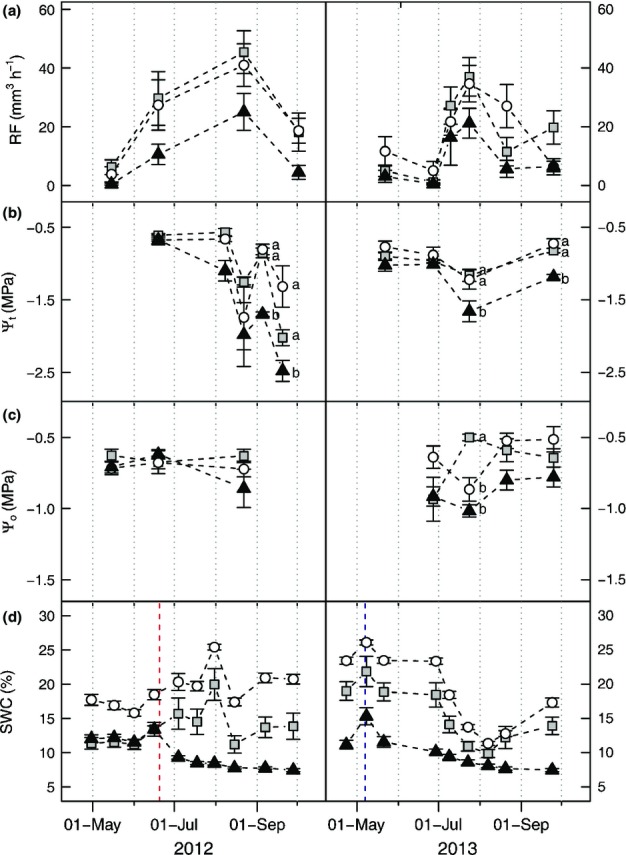
Mean ± SE of tree physiological parameters measured on the 18 Norway spruce (*Picea abies*) sample trees and of soil moisture at the experimental site in the seasons 2012 and 2013 by date and treatment (C, control plots; circles; SC, semicover plots; squares; FC, full-cover plots; triangles). (a) Resin flow (RF) rate recorded within 12 h overnight. (b) Pre-dawn twig water potential (Ψ_t_, MPa) determined in the field directly after sampling of twigs using a shotgun. Measurements on 19 June 2012 were made for one tree per treatment on twigs cut from the crown. (c) Osmotic potential at full saturation (Ψ_o_, MPa) of secondary phloem. Sampling was performed on the specified dates, with measurement later in the laboratory. (d) Volumetric soil water content (SWC). The red line marks the time of roof establishment in June 2012, and the blue line indicates the second irrigation event in spring 2013. Different letters within the same date indicate significant differences between the treatments (Scheffé post hoc tests;*P < *0.05).

SWC conditions were similar for 0–30 cm and 0–60 cm soil depth; therefore we only present the data relating to the upper, main rooting horizon (Fig.3d). As expected, SWC was significantly lowest for the FC treatment and highest for the control (effect of treatment:*F *= 94.114, df = 2;*P *< 0.001). Date had a significant effect (Wilks’ lambda;*P *= 0.001), as well as the interaction of treatment and date (*P *< 0.001). In 2013, SWC remained significantly lowest for FC from 18 July until the end of the season (24 September). As a consequence of pronounced heat and drought in this year, SWC was significantly reduced for all treatments, and SC and C plots were equally dry in August 2013. A clear increase in SWC from the end of the season 2012 to spring 2013 was recorded for all treatments, as a result of snow accumulated in C and SC plots and irrigation of FC. Irrigation led to a rise in soil moisture comparable to SWC values before roof establishment. This increase remained significant until June 2013, when the warm and dry weather conditions eventually reduced SWC to values obtained after sheltering.

### Effects of drought treatment on tree physiological indicators of stress and resistance to*I. typographus*

Treatment-induced drought stress of the sample trees was reflected by significantly lower shoot lengths of FC compared with SC and C trees (effect of treatment:*F* ratio = 12.275, df = 2;*P *= 0.012) and significantly shorter needles of FC compared with C trees (effect of treatment, without September dates:*F* ratio = 8.056, df = 2;*P *= 0.027) in 2013 (Table S1).

RWC of phloem and sapwood of the target trees was significantly affected by date (Wilks’ lambda: phloem*P *< 0.005; sapwood*P *< 0.001), but not by the interaction of treatment and date (phloem*P *= 0.211; sapwood*P *= 0.157). RWC of the sapwood continuously decreased from June (FC 77%, SC 83%, and C 79%) to September 2013 (FC 61%, SC 66%, and C 59%), while mean phloem RWC was more variable between recording dates and treatments, and did not follow a trend. Treatment generally had no effect (phloem:*F* ratio = 3.382, df = 2;*P *= 0.061; sapwood:*F* ratio = 3.231, df = 2;*P *= 0.068), except for 27 August 2012 (phloem:*F* ratio = 7.352, df = 2;*P *= 0.006; sapwood:*F* ratio = 5.713, df = 2;*P *= 0.014) with significantly lower values for SC and FC (Fig. S2).

Ψ_o_ was not significantly affected by date (Wilks’ lambda:*P *= 0.13), but by the interaction of treatment and date (*P *= 0.001). Analysing each date separately, Ψ_o_ was lowest for FC trees in August 2012, and July, August and September 2013 (Fig.3c), but differences between the treatments were significant only in July 2013 between FC and C compared with SC (*F* ratio = 23.09, df = 2,*P *< 0.001).

Ψ_t_ was significantly affected by date (Wilks’ lambda:*P *< 0.001) and the interaction of treatment and date (*P *< 0.001) (Fig.3b). C and SC trees did not differ significantly on any recording date, but FC trees displayed significantly lower values on 8 August (*F* ratio = 10.214, df = 2;*P *= 0.002) and 5 September 2012 (*F* ratio = 70.255, df = 2;*P *< 0.001) as well as on 24 July (*F* ratio = 4.902, df = 2;*P *= 0.023) and 25 September 2013 (*F* ratio = 26.913, df = 2;*P *< 0.001). In 2013, Ψ_t_ was significantly correlated with SWC (*r *= 0.685;*P *= 0.014;*n *= 12), and the summer drought event led to significantly lower Ψ_t_ for all treatments on 24 July 2013 (*F* ratio = 12.429, df = 3;*P *< 0.001).

**Figure 4:**
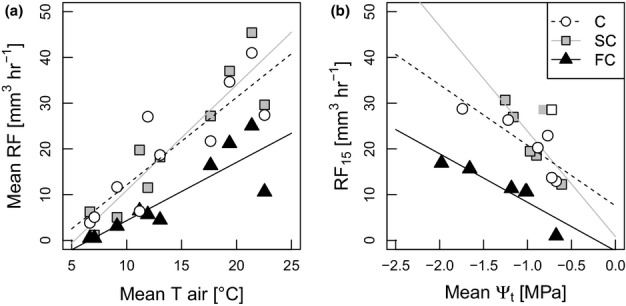
Linear relationships within treatments of (a) mean resin flow (RF) rate and mean temperature during recording time (C, control plots; circles;*r*^2^ =* *0.75;*P < *0.01; SC, semicover plots; squares;*r*^2^ = 0.85;*P *< 0.001; FC, full-cover plots; triangles;*r*^2^ = 0.74;*P *< 0.01;*n *= 10) and (b) mean rates of RF standardized at 15°C (RF_15_) for all recording dates and mean pre-dawn twig water potential (Ψ_t_) (C,*r*^2^ = 0.70;*P *< 0.05; SC,*R*^2^ = 0.57; not significant; FC,*r*^2^ = 0.82;*P *< 0.05;*n *= 10) for Norway spruce (*Picea abies*) sample trees.*T*_air_, air temperature.

RF was significantly affected by date (Wilks’ lambda:*P *= 0.002), but not by the interaction of treatment and date (*P *= 0.660), although mean RF values of FC were consistently lower than those of C and SC (Fig.3a). For all treatments, RF was highest in August 2012 and again in July 2013, with the lowest values in May 2012 and June 2013, respectively. This dynamic was probably driven by temperature, with mean treatment RF highly correlated with mean temperature during sampling time (Fig.4a). Mean rates of RF_15_ were significantly correlated to mean Ψ_t_ of the FC and C treatments (Fig.4b). Except for September 2013 measurements, RF records were significantly correlated between the dates (e.g. October 2012/May 2013:*r *= 0.625;*P *= 0.006; May/August 2013:*r *= 0.793;*P *< 0.001;*n *= 18). Variation in RF_15_ of each sample tree was nevertheless high between the recording dates (coefficient of variation: C: 0.51 in 2012 and 0.56 in 2013; SC: 0.63 in 2012 and 0.46 in 2013; FC: 0.90 in 2012 and 0.74 in 2013). RF intensity generally decreased in 2013 compared with 2012 values (0.83-fold decreases in maximum values and 0.64-fold decreases in minimum values; similar for all treatments). RF was correlated with the number of resin canals (May 2013:*r *= 0.553;*P *= 0.017;*n *= 18; August 2013:*r *= 0.536;*P *= 0.022;*n *= 18), with the total area of resin canals (May 2013:*r *= 0.596;*P *= 0.015;*n *= 16), and with the number of epithelial cells (May 2013:*r *= 0.536;*P *= 0.027;*n *= 17; August 2013:*r *= 0.385; not significant;*n *= 18). ANOVA showed no significant differences in bark anatomical parameters between treatments on either sampling date (Table S2).

**Figure 5:**
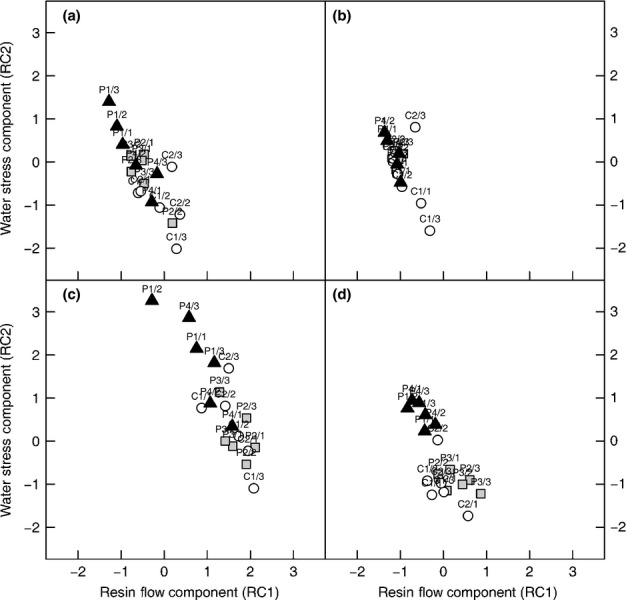
Component scores of the individual sample trees as calculated from a principal components analysis with subsequent component reduction and rotation. The Norway spruce (*Picea abies*) sample trees are plotted separately for period 1 (a), period 2 (b), period 3 (c), and period 4 (d) and labelled by their code name, with component scores marked by circles, squares or triangles according to treatment (C, control plots; SC semicover plots; FC, full-cover plots).

From the PCA, the first (eigenvalue 2.94) and second (eigenvalue 0.69) principle components together accounted for 91% of the variance in the data (Fig. S3a). While the transformed values of mean air temperature and maximum vapour pressure deficit loaded significantly on both rotated components, the loading pattern of the transformed values of RF (RC1 0.91; RC2 0.06) and Ψ_t_ (RC1 0.23; RC2 0.96; Fig. S3b) essentially allowed each sample tree for a given measurement date to be positioned in a two-dimensional RF (RC1)–water stress (RC2) space. Over each of the four periods, the trees of each treatment tended to overlap with one another, again demonstrating the inconsistent treatment effect on RF and Ψ_t_ (Fig.5). Nonetheless, by looking at the periods separately (i.e. removing temperature influence), a negative correlation between the water stress and RF components emerged. Finally, it was interesting to note that tree C2/3 tended to separate itself from the other control trees during periods 1, 2 and 3.

### Bark beetle attack experiments

**Figure 6:**
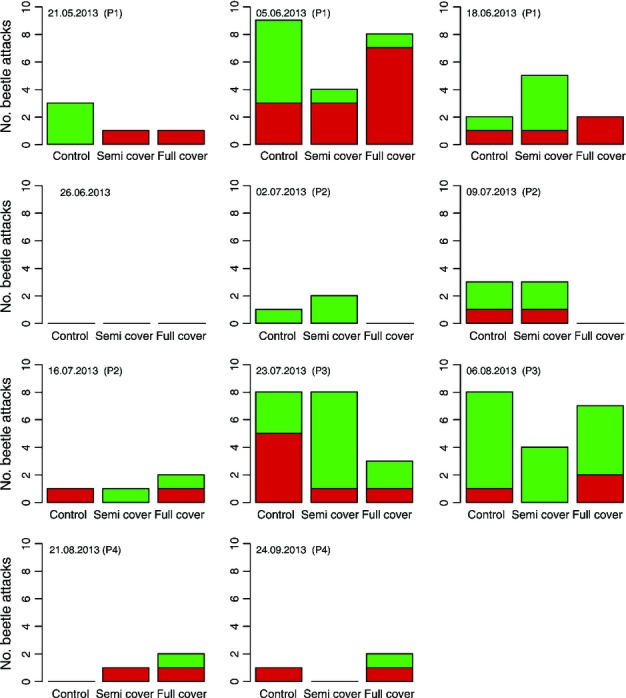
Number of successful (Suc; red bars) and defended (Def; green bars) attacks by the Eurasian spruce bark beetle (*Ips typographus*) on Norway spruce (*Picea abies*) sample trees by treatment and date (day.month.year). Single dates were pooled into the periods: (1) spring, (2) early summer, (3) mid-summer, and (4) late summer and autumn. Drought treatment significantly impacted proportions of successful and defended attacks (χ^2^* *= 6.249, df = 2;*P *= 0.044), but differences in attack numbers between the treatments were not significant.

Among recollected and sexed individuals (*n *= 2453), males slightly outbalanced females in June (56%), July (51%) and August (52%), but not in September (49%). Predominantly males attacked the trees, but equal proportions of males and females were found within the attack boxes and in the exit devices. Air temperature maxima during the attack experiments ranged between 15.9°C on 26 June and 33.4°C on 6 August 2013. Beetles were inactive on the first occasion, but showed activity during all other attack experiments (maximum temperatures ≥ 16.3°C). Maximum temperature positively affected the mean proportions of beetles leaving the initial bottle (*r*^2^ = 0.73;*P *< 0.001;*n *= 12) and exiting the attack box (*r*^2^ = 0.84;*P *< 0.001;*n *= 12) (Fig. S4). Overall, infestation was low, with ≤ 5 beetles attacking one tree in any one experiment (92 attacks in total). Drought treatment significantly influenced proportions of successful and defended attacks over the season (χ^2^ = 6.249, df = 2;*P *= 0.044). While attacks were most numerous on C trees (*n *= 36) with most of them defended (*n *= 23), FC trees exhibited the lowest number of attacks (*n *= 27), but many of them were successful (*n *= 16). Attack activity was higher in early spring (*n *= 35) and mid-summer (*n *= 38), and low in early summer (*n* = 13), late summer and autumn (*n *= 6) (Fig.6). The differences between the periods in the mean sum of attacks per recording date were significant (*F *= 8.449, df = 3;*P *< 0.001). Fifty-four per cent of attacks were successful in spring, but only 26% in mid-summer.

### Tree physiological indicators of drought stress related to attack by*I. typographus*

Within the periods, we found a positive relationship of RF with the number of defended attacks in spring (RF, 22 May:*r *= 0.468;*P *≤ 0.050;*n *= 18) and with the number of successful attacks in early summer (RF, 27 June:*r *= 0.687;*P *= 0.002;*n *= 18). Treatment had a significant impact on the proportion of successful and defended attacks in spring (χ^2^ = 9.750, df = 2;*P *= 0.008), but not in the other periods. The measured tree physiological variables were significant determinants of defended and successful attack ratios in all periods except for late summer and autumn. The proportion of defended attacks decreased with more negative Ψ_t_ in periods 1–3 and increased with RF in spring (see model coefficients and*P* values of the respective variables in Table2). Lower rates of RF and increasingly negative Ψ_t_, in contrast, promoted successful attacks in spring and early summer. In mid-summer, the proportion of successful attacks increased with more negative Ψ_o_, although this was not significant.

**Table 2:** Results of the generalized linear models for proportions of defended (def) and successful (succ) attacks, and the sum of attacks by the Eurasian spruce bark beetle (*Ips typographus*) on Norway spruce (*Picea abies*) trees in the periods: (1) spring, (2) early summer, (3) mid-summer and (4) late summer and autumn

		Model			Parameter		
		χ^2^	df	*P*	var	coeff	*P*
Period 1	**def**	167.476	2	**< 0.001**	**ψ_t_**	**1.807**	**< 0.001**
					**RF**	**0.043**	**< 0.001**
					**const**	**4.817**	**< 0.001**
	**succ**	14.496	2	**≤ 0.001**	ψ_t_	−0.377	0.061
					**RF**	−**0.016**	**0.002**
					**const**	**3.317**	**< 0.001**
	sum	0.921	2	0.631	ψ_t_	0.133	0.877
					RF	0.018	0.327
					const	0.653	0.412
Period 2	**def**	202.096	4	**< 0.001**	**ψ**_**t**_	**3.080**	**< 0.001**
					**RF_june**	−**0.063**	**< 0.001**
					**RF_july**	**0.027**	**< 0.001**
					**ψ_o_**	−**0.697**	**< 0.001**
					**const**	**5.398**	**< 0.001**
	**succ**	321.378	4	**< 0.001**	ψ_t_	−0.605	0.272
					**RF_june**	**0.200**	**< 0.001**
					**RF_july**	−**0.060**	**< 0.001**
					ψ_o_	0.424	0.356
					**const**	**2.330**	**< 0.001**
	sum	2.710	4	0.607	ψ_t_	1.149	0.524
					RF_june	0.078	0.216
					RF_july	0.008	0.681
					ψ_o_	0.185	0.878
					const	0.483	0.741
Period 3	**def**	71.650	3	**< 0.001**	**ψ**_**t**_	**0.764**	**< 0.001**
					**RF**	−**0.006**	**0.006**
					ψ_o_	−0.139	0.248
					**const**	**5.221**	**< 0.001**
	succ	3.629	3	0.304	ψ_t_	0.058	0.715
					RF	0.003	0.373
					ψ_o_	−0.403	0.074
					const	2.634	< 0.001
	sum	7.534	3	0.057	**ψ**_**t**_	**1.176**	**0.027**
					RF	−0.023	0.059
					ψ_o_	−0.226	0.732
					**const**	**2.767**	**0.002**
Period 4	no attack	7.709	3	0.052	ψ_t_	2.163	0.484
					RF	0.191	0.210
					ψ_o_	5.708	0.205
					const	4.990	0.088

Significant models and parameters are shown in bold.

**Figure 7:**
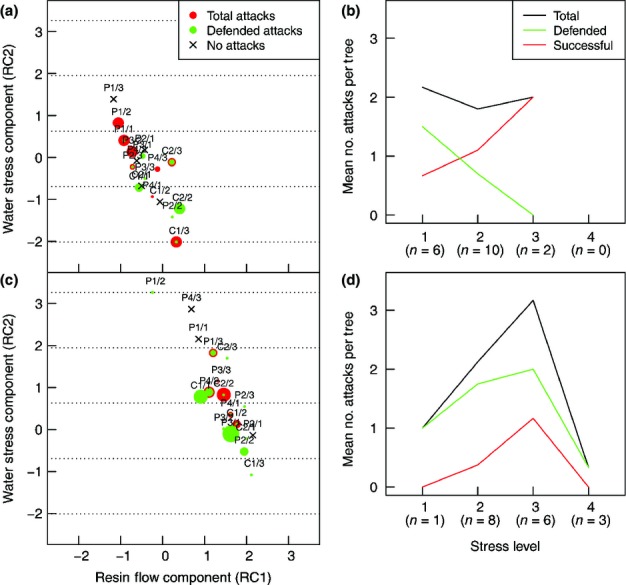
Component scores of the individual Norway spruce (*Picea abies*) sample trees as calculated from a principal components analysis with subsequent component reduction and rotation plotted separately for period 1 (a) and period 3 (c). The sample trees are labelled with their code name, with the component scores marked by a cross, where no bark beetle (*Ips typographus*) attack was observed, or by a circle, if attack was recorded during the respective period. Red circles, total attacks; green circles, the proportion of attacks that were defended. The diameter of the circles corresponds to the respective numbers of attacks. Four water stress levels are delineated by horizontal dotted lines, with the mean number of total, successful and defended bark beetle attacks per stress level plotted on separate graphs for period 1 (b) and period 3 (d). Below the*x*-axis, the number (*n*) of sample trees per stress level is given in parentheses.

To investigate general trends in tree defence and host acceptance, the sample trees in periods 1 and 3 were binned into four stress levels based on the observed range (−2.01 to 3.27) in the water stress component (RC2) (Fig.7a,c), with mean numbers of total, successful and defended attacks per tree calculated (Fig.7b,d). In both periods, the proportion of defended attacks generally declined over the first three stress levels, while the percentage of mean total attacks per tree that were successful increased. The highest stress level was only relevant for period 3 (Figs5, S5), although the average number of total attacks per tree and the ratio attacked : nonattacked trees for the fourth stress level were lower than at any other stress level (Fig.7c,d).

## Discussion

Extreme climatic events such as heat waves and extended droughts are likely to become more intense and frequent over the next few decades (IPCC, 2013), potentially amplifying the risks of insect attack and tree mortality in forest ecosystems adapted to temperate growth conditions (Hart*et al*., 2014). Here, we report on the first results of the Rosalia Roof Project, a comprehensive study of how drought-induced changes in physiology affect the predisposition of Norway spruce to attack by the Eurasian spruce bark beetle. Essentially, we found that tree water status and RF were significantly influenced by soil water supply, but also highly variable over two seasons of experimental drought in response to weather conditions. Observed attack patterns indicate seasonal fluctuations in predisposition, with decreasing pre-dawn twig water potentials causing reductions in resistance against attack. The data, however, indicate that, past a certain extreme stress threshold, host acceptance by*I. typographus* is reduced despite the weakened defence systems of these potential hosts.

### Effects of treatment on tree physiological indicators of stress and resistance

The physiological parameters monitored over 2 yr of drought manipulation demonstrated both expected and unexpected temporal and between-treatment patterns. Although showing significant differences between C and roofed trees (FC and SC) in August 2012, the RWC of phloem and sapwood was less related to water availability in the second year. Apart from the common seasonal decline in wood water content, we would have anticipated a stronger reduction of stem water in the trees fully deprived of water (Kravka*et al*., 1999) and particularly in response to the extremely hot and dry weather conditions of summer 2013 (Figs2, S2). Instead, effects of treatment were clearly indicated by twig water potential and osmotic potential of the phloem soon after roofing (Fig.3b,c). Water resources were restocked because of extended snow cover and irrigation, leading to physiological recovery of all sample trees until spring 2013. The following period of extreme heat and drought in July 2013, however, provoked a distinct reduction in soil water content, which was similar for all treatments and associated with increased stress, especially for the FC trees, but also even for the C trees (Figs3b–d, 5c). In line with earlier studies, we recorded pre-dawn twig water potentials ranging from −4.15 MPa (August 2012 for FC) in the case of severe water stress to values of −0.55 to −0.85 MPa for unstressed trees and before roof establishment (Lu*et al*., 1995; Rothe*et al*., 2002; Branco*et al*., 2010). Interestingly, despite intense summer heat and drought, pre-dawn water potentials only reached a minimum value of −2.8 MPa in 2013.

Our findings regarding constitutive RF correspond to the known seasonal patterns and strong positive correlation with temperature (Baier*et al*., 2002; Gaylord*et al*., 2007; Knebel*et al*., 2008). As found in other studies, we also observed large variation in flow rates between trees (Christiansen & Horntvedt, 1983; Schroeder, 1990) and a strong relationship of RF to the density of resin ducts and epithelial cells (Baier*et al*., 2002; Rosner & Hannrup, 2004). The size and number of radial resin canals did not vary significantly between the treatments on either sampling date (Table S2), because of the longer time frame over which Norway spruce adapts these strongly inherent characteristics to changes in water supply (Rosner & Hannrup, 2004). Resin exudation, however, varied significantly over time, with the severely stressed FC trees generally demonstrating the lowest RF rates (Figs3a, 4a). Our observations corroborate the assumption of decreased water and/or carbon resources available for resin production in the most severely drought-stressed trees (Lombardero*et al*., 2000; Gaylord*et al*., 2013), which also showed significant reductions in shoot and needle growth (Table S1). However, the partially stressed SC trees did not show higher constitutive RF, as may have been expected according to Herms & Mattson (1992). Interestingly, however, mean temperature-standardized RF within each treatment was clearly enhanced by more negative twig water potentials (Fig.4b) despite the apparent negative correlation between the water stress and RF components (Fig.5). This discrepancy may be attributable to the fact that both FC treatments showed lower soil water contents even before the roof construction (Fig.3d) and/or a differential reaction of tree defence systems to chronic and acute stress. We nevertheless recorded lowered resin exudation in the second year of the experiment for all treatments, which rules out any artificial induction of RF attributable to lesions caused by sampling (Knebel*et al*., 2008; Gaylord*et al*., 2011).

### Attack by*I. typographus*

In contrast to one-way choice experiments and the use of pheromone dispensers (Bakke, 1983; Raffa & Berryman, 1983; Mulock & Christiansen, 1986; Lorio*et al*., 1995; Baier, 1996; Turčáni & Nakládal, 2007), our attack boxes neither forced nor stimulated attack, nor resulted in immediate mass attack of the sample trees. Instead, controlled, simultaneous observation of both beetle behaviour, when presented with a potential host, and host reaction to attack was possible with this experimental approach. We were thus able to evaluate host acceptance by the beetles (sum of attacks) and the defence capability of the trees (ratio of successful and defended attacks) under controlled beetle densities. As expected for living trees and low beetle pressure, the total number of attacks remained low over the study (Mulock & Christiansen, 1986; Boone*et al*., 2011). Interestingly, attack peaks corresponded to periods of spring and summer flight in the natural environment (Figs6, S6), despite the experimental manipulation of host selection and timing of contact between beetles and trees. These dynamics in beetle activity were probably driven by temporal variation in both chemical stimuli and air temperature. Trees are most attractive for swarming beetles after winter as a consequence of the mobilization of water and nutrients and probable high emission of volatile attractants (Baier*et al*., 2002), explaining the high number of total attacks observed in spring across all treatments. Following a period of low attack in early summer, boring numbers again increased in mid-summer for all treatments. The higher temperatures during this period acting upon the composition and concentration of released monoterpenes probably added to the attractiveness of all sample trees for attack (Baier & Bader, 1997; Hietz*et al*., 2005; Branco*et al*., 2010). Temperature directly affects ectothermic organisms; hence we observed most movements in and out of the attack boxes and even boring attempts of the beetles in cases of temperatures above 20°C (Fig. S4). Beetles stopped exiting the start bottles below air temperatures of 16.0°C, which corresponds to the known temperature thresholds for flight (Lobinger, 1994; Wermelinger, 2004). Despite sufficiently high temperature, general beetle activity and attack ceased in late summer and autumn, possibly as a result of reduced activity under shortening day-lengths (Baier*et al*., 2007) and/or a seasonal reduction in olfactory cues from the trees.

### Tree physiological indicators of drought stress related to host acceptance by*I. typographus* and tree resistance to attack

Pre-dawn twig water potential, a commonly used drought stress proxy (Sellin, 1997), was used to assess the stress status of the sample trees over the study (Figs S3b, 5). Drought stress varied between and within the treatments, and between seasonal periods. While total retention of rainwater provoked increased water stress and decreased RF for FC trees over all periods, physiological states of SC and C trees were less stable. Drought stress of C trees was predominantly low in spring and again in autumn, but for some individuals increased to higher levels in response to the drought period in mid-summer. An outlier (C2/3) already exhibited stronger negative twig water potentials and above-average resin exudation in spring and early summer. This was an interesting observation in view of the fact that we identified heavy infection of this tree by*Armillaria* sp., a pathogenic fungus which has been reported to enhance drought stress and bark beetle infestation in Eastern European Norway spruce forests (Jakus, 2001; Grodzki, 2007). We also recorded pronounced but predominantly defended attack for C2/3. Indeed, although C trees in general were able to prevent most of the attacks in spring by resin exudation, they were nonetheless just as attractive to the beetles (Fig.6). It is well known that terpenes as major compounds of resin are specifically relevant for primary attraction of bark beetles, although high concentrations can inhibit attack (Zhao*et al*., 2011; Raffa, 2014). The main component of spruce resin, (-)-α-pinene, for instance, is used for pheromone synthesis despite its toxicity to bark beetles in high concentrations (Erbilgin*et al*., 2003, 2007). Moreover, increased total monoterpene contents and changes of relative proportions of specific compounds in the bark of host trees in response to attack are recognized as significant determinants of further colonization success (Boone*et al*., 2011; Schiebe*et al*., 2012).

Rather than treatment*per se*, variation in the stress status of the individual trees appeared to be the important factor driving predisposition. For instance, on 23 July the C trees were actually the least successful in defending against beetle invaders, despite high RF rates (Figs3a, 6). This observation was probably attributable to the drought period at the time, indicated by the relatively low twig water potentials and migration of these trees into higher stress states (Fig.5c). While a clear correlation of increased RF with higher proportions of defended attacks was found only in spring, decreasing pre-dawn twig water potential significantly reduced the defence capability of trees across all treatments from spring to mid-summer. Osmotic potential generally proved a less reliable indicator of resistance (Table2). However, our results do not support a monotonic relationship between water stress, as indicated by pre-dawn twig water potential, and predisposition to attack by*I. typographus*. While it is likely that the ability to defend against bark beetles is reduced, our data indicate that with increasingly severe stress host trees are not necessarily invaded, as they become less attractive to*I. typographus* (Fig.7c,d).

### Implications

Despite the potential triggering effects of precipitation deficits, concrete evidence linking drought-induced physiological stress of trees and their predisposition to bark beetle attack is still lacking. We provide empirical data corroborating the impact of chronic and acute drought stress on host acceptance by*I. typographus* and the defence capability of Norway spruce. Higher proportions of prevented attacks were observed among trees exhibiting low water stress and high RF, with successful attacks increasing with tree water stress. However, while severely weakened Norway spruce might be easily conquered by*I. typographus*, our results indicate that such trees may escape attack as a result of reduced host acceptance. Nevertheless, the weakened trees may still represent an attractive host for other, secondary scolytid species as presumed by the growth–differentiation balance hypothesis. We suggest further studies under conditions of extended and intensified drought, including additional analyses of other physiological processes relevant to predisposition, for example hydraulic failure and depletion of nonstructural carbohydrates (McDowell*et al*., 2008), as well as induced defence reactions such as changes in emissions of volatile organic compounds.

## Supporting Information

**Fig. S1**‘Attack box’ developed to experimentally test the predisposition of the Norway spruce (*Picea abies*) sample trees to infestation by the Eurasian spruce bark beetle,*Ips typographus*. **Fig. S2** Mean ± SE of relative water content (RWC) of phloem and xylem of Norway spruce (*Picea abies*) sample trees. **Fig. S3** Results from principal components analysis: the variance explained by each of the four principal components and bi plot showing the component scores for the first (RC1) and second (RC2) rotated components. **Fig. S4** Linear relationships of maximum temperature and proportions of bark beetles (*Ips typographus*) leaving the start box and entering the exit box. **Fig. S5** Component scores of the individual Norway spruce (*Picea abies*) sample trees as calculated from a principal components analysis with subsequent component reduction and rotation. Circles represent total attacks and proportion of defended attacks by the Eurasian spruce bark beetle (*Ips typographus*). **Fig. S6** Number of bark beetles (*Ips typographus*) caught in a pheromone baited trap located near the climate station in a clearing 200 m from the experimental site. **Table S1** Mean ± SE of shoot and needle lengths of Norway spruce (*Picea abies*) sample trees subject to different drought treatments **Table S2** Mean ± SE of the bark anatomical parameters of Norway spruce (*Picea abies*) sample trees subject to different drought treatments
