# Supplementary material for: Do water-limiting conditions predispose Norway spruce to bark beetle attack?
Source: New Phytol. 2014 Nov 21;205(3):1128–41. doi: 10.1111/nph.13166 (PMC4315866; doi:10.1111/nph.13166)
Supplement: Supplementary file 1 — Fig. S1‘Attack box’ developed to experimentally test the predisposition of the Norway spruce (Picea abies) sample trees to infestation by the Eurasian spruce bark beetle,Ips typographus. Fig. S2 Mean ± SE of relative water content (RWC) of phloem and xylem of Norway spruce (Picea abies) sample trees. Fig. S3 Results from principal components analysis: the variance explained by each of the four principal components and bi plot showing the component scores for the first (RC1) and second (RC2) rotated components. Fig. S4 Linear relationships of maximum temperature and proportions of bark beetles (Ips typographus) leaving the start box and entering the exit box. Fig. S5 Component scores of the individual Norway spruce (Picea abies) sample trees as calculated from a principal components analysis with subsequent component reduction and rotation. Circles represent total attacks and proportion of defended attacks by the Eurasian spruce bark beetle (Ips typographus). Fig. S6 Number of bark beetles (Ips typographus) caught in a pheromone baited trap located near the climate station in a clearing 200 m from the experimental site. Table S1 Mean ± SE of shoot and needle lengths of Norway spruce (Picea abies) sample trees subject to different drought treatments Table S2 Mean ± SE of the bark anatomical parameters of Norway spruce (Picea abies) sample trees subject to different drought treatments [file nph0205-1128-sd1.pdf]

New Phytologist Supporting Information

Article title: **Do water limiting conditions impair defence capability of Norway spruce against bark beetle attack?**

Authors: Sigrid Netherer, Bradley Matthews, Klaus Katzensteiner, Emma Blackwell, Patrick Henschke, Peter Hietz, Josef Pennerstorfer, Sabine Rosner, Silvia Kikuta, Helmut Schume and Axel Schopf

Article acceptance date: 08 October 2014

The following Supporting Information is available for this article:

Fig. S1 'Attack box' developed to experimentally test predisposition of the Norway spruce (*Picea abies*) sample trees to infestation by the Eurasian spruce bark beetle (*Ips typographus*). The boxes were attached to wooden frames permanently fixed on the sample trees fastened by belts to be closed during the course of each attack experiment.

Fig. S2 Mean ± SE of relative water content (RWC, %) of phloem and xylem of Norway spruce (*Picea abies*) recorded in periodic intervals on the sample trees during the seasons 2012 and 2013; treatments within the same date not labelled by the same letter are significantly different ($P<0.05$).

Fig. S3 Results from principal components analysis: (a) the variance explained by each of the four principal components and (b) bi plot showing the component scores for the first (RC1) and second rotated components (RC2) of each Norway spruce (*Picea abies*) sample tree for each period (black circles), and the component loadings (upper *x*-axis, right *y*-axis, red arrows) of the four transformed variables (pre-dawn twig water potential (tWP), mean air temperature (T air), maximum vapor pressure deficit (VPD), and resin flow (RF)).

Fig. S4 Linear relationships of maximum temperature measured in the course of the attack experiments and proportions of bark beetles (*Ips typographus*) leaving the start box (r^2=0.73, $P<0.001$) and proportions of beetles entering the exit box (r^2=0.84, $P<0.001$).

Fig. S5 Component scores of the individual Norway spruce (*Picea abies*) sample trees as calculated from a principal components analysis with subsequent component reduction and

rotation. The samples trees are plotted separately for period 1 (a), period 2 (b), period 3 (c), and period 4 (d). The sample trees are labelled with their code name, with the component scores marked by a cross in the case where no bark beetle (*Ips typographus*) attack was observed or by a circle if attack was recorded during the respective period. Red circles represent total attacks, with green circles representing the proportion of attacks which were defended. The diameter of the circles corresponds to the respective numbers of attacks.

Fig. S6 Number of bark beetles (*Ips typographus*) caught in a pheromone baited trap located near the climate station on a clearing 200 m from the experimental site. The pheromone trap was emptied every 1 to 2 wk, with mean catch rate calculated by dividing the total number of caught beetles by the number of days since the trap was last emptied.

Table S1 Mean ± SE of shoot and needle lengths (mm) measured on Norway spruce (*Picea abies*) twigs sampled between June and September 2013 by treatment (FC, fullcover; SC, semicover; C, control). Significant differences between the treatments are labelled by different letters (shoot lengths, *F ratio=12.275,* df=2, *P*=0.012; needle lengths, *F* ratio=8.056, df=2, *P*=0.027).

Table S2 Mean ± SE of the bark anatomical parameters of Norway spruce (*Picea abies*) sample trees: number of resin canals (no. RC, cm^{-2}), total area of resin canals cm^{-2}, and number of epithelial cells (no. EC, cm^{-2}). The bark anatomical parameters do not differ significantly between treatments on any sampling date.

Fig. S1 ‘Attack box’ developed to experimentally test predisposition of the Norway spruce (*Picea abies*) sample trees to infestation by the Eurasian spruce bark beetle (*Ips typographus*). The boxes were attached to wooden frames permanently fixed on the sample trees fastened by belts to be closed during the course of each attack experiment.

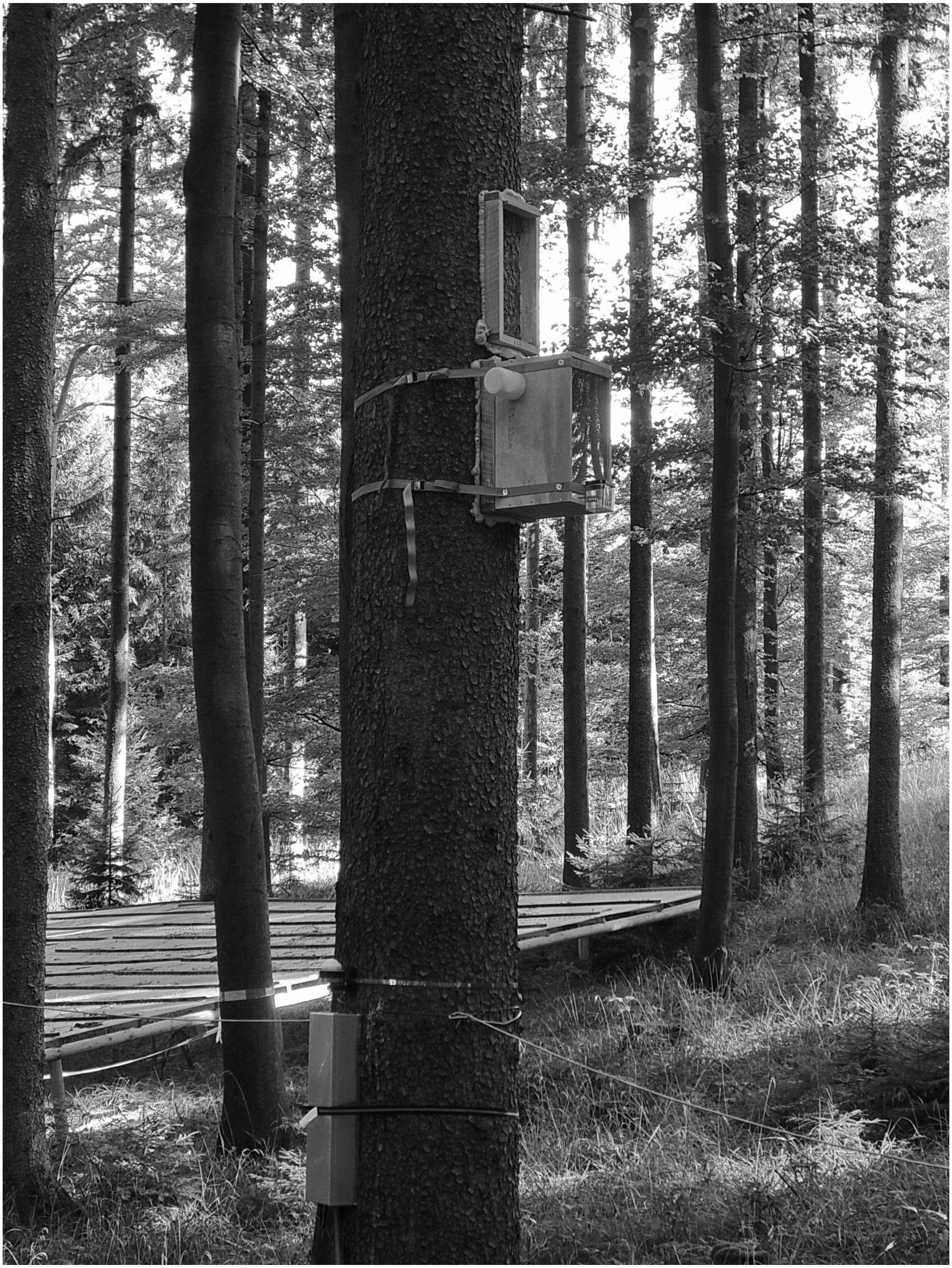

Fig. S2 Mean ± SE of relative water content (RWC, %) of phloem and xylem of Norway spruce (*Picea abies*) recorded in periodic intervals on the sample trees during the seasons 2012 and 2013; treatments within the same date not labelled by the same letter are significantly different (P<0.05).

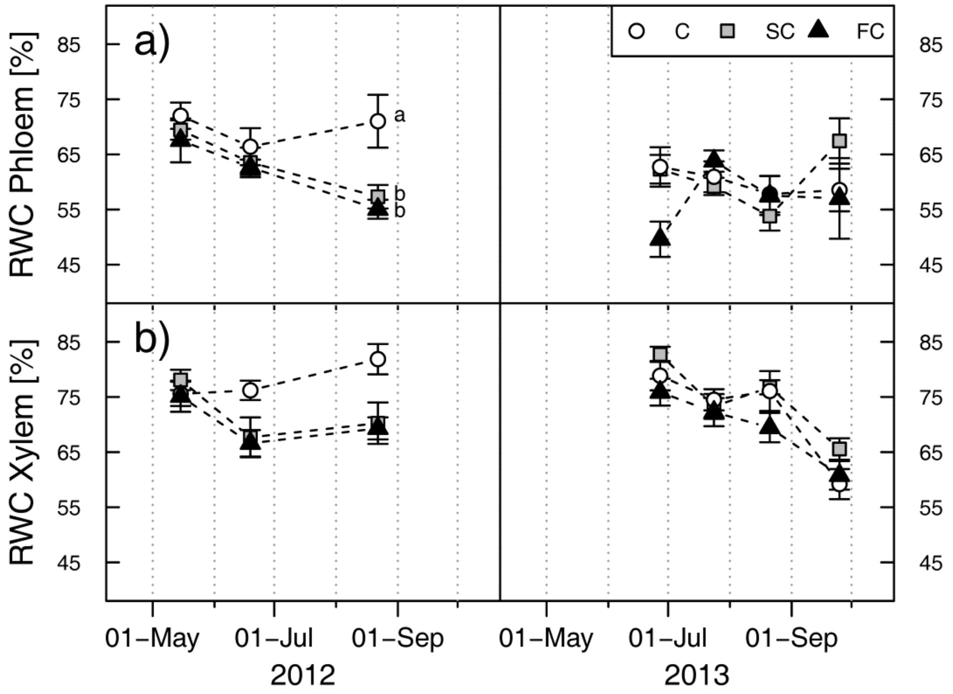

Fig. S3 Results from principal components analysis: (a) the variance explained by each of the four principal components and (b) bi plot showing the component scores for the first (RC1) and second rotated components (RC2) of each Norway spruce (*Picea abies*) sample tree for each period (black circles), and the component loadings (upper *x*-axis, right *y*-axis, red arrows) of the four transformed variables (pre-dawn twig water potential (tWP), mean air temperature (T air), maximum vapor pressure deficit (VPD), and resin flow (RF)).

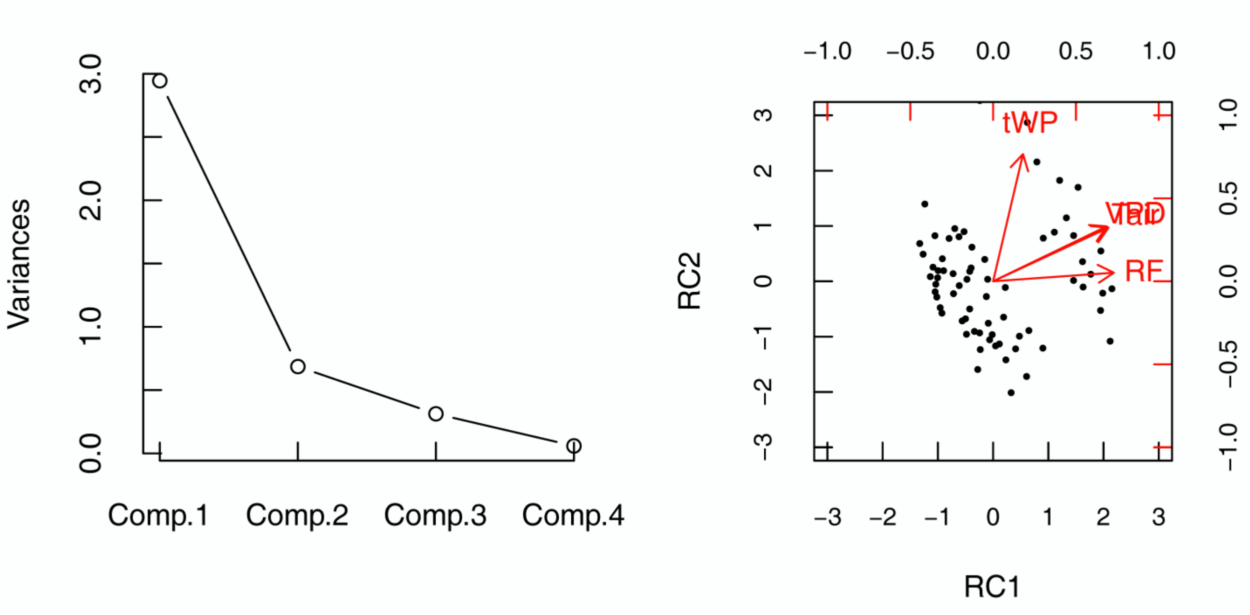

Fig. S4 Linear relationships of maximum temperature measured in the course of the attack experiments and proportions of bark beetles (*Ips typographus*) leaving the start box (r^2=0.73, P<0.001) and proportions of beetles entering the exit box (r^2=0.84, P<0.001).

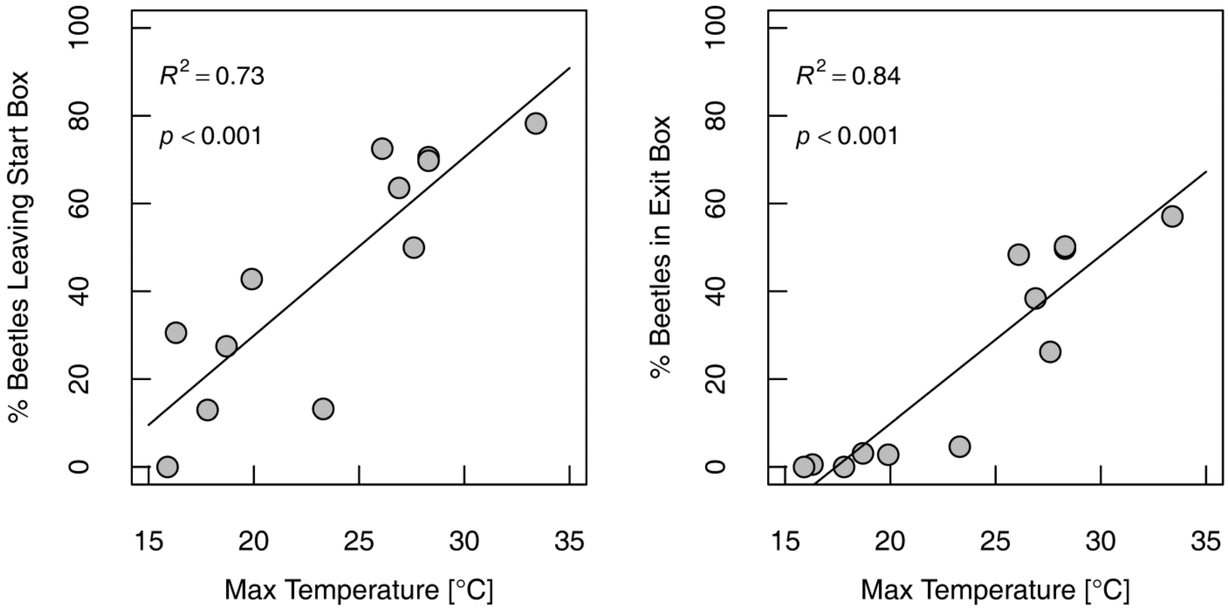

Fig. S5 Component scores of the individual Norway spruce (*Picea abies*) sample trees as calculated from a principal components analysis with subsequent component reduction and rotation. The samples trees are plotted separately for period 1 (a), period 2 (b), period 3 (c), and period 4 (d). The sample trees are labelled with their code name, with the component scores marked by a cross in the case where no bark beetle (*Ips typographus*) attack was observed or by a circle if attack was recorded during the respective period. Red circles represent total attacks, with green circles representing the proportion of attacks which were defended. The diameter of the circles corresponds to the respective numbers of attacks.

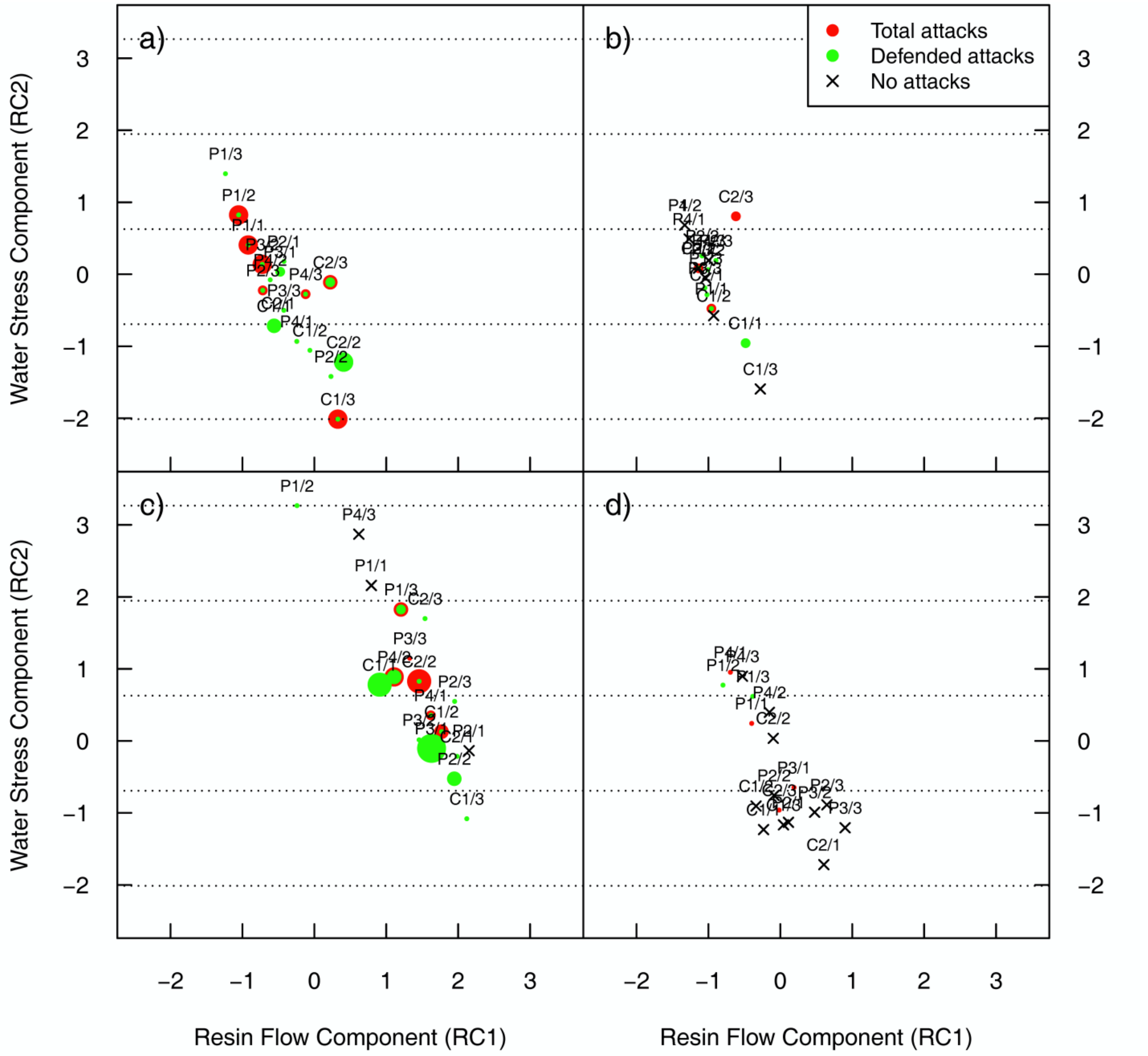

Fig. S6 Number of bark beetles (*Ips typographus*) caught in a pheromone baited trap located near the climate station on a clearing 200 m from the experimental site. The pheromone trap was emptied every 1 to 2 wk, with mean catch rate calculated by dividing the total number of caught beetles by the number of days since the trap was last emptied.

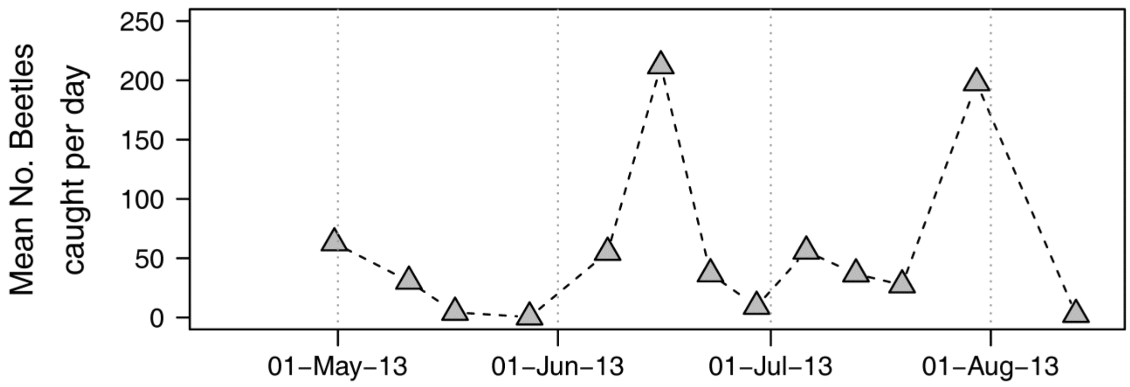

Table S1 Mean ± SE of shoot and needle lengths (mm) measured on Norway spruce (*Picea abies*) twigs sampled between June and September 2013 by treatment (FC, fullcover; SC, semicover; C, control). Significant differences between the treatments are labelled by different letters (shoot lengths, *F* ratio=12.275, df=2, *P*=0.012; needle lengths, *F* ratio=8.056, df=2, *P*=0.027).

	Shoot lengths				Needle lengths			
	n	mean	SE		*n*	mean	SE	
FC	15	30.50	3.23	a	12	14.39	0.96	a
SC	15	44.63	3.16	b	10	18.55	0.85	b
C	13	44.73	4.61	b	10	16.02	0.88	b

Table S2 Mean ± SE of the bark anatomical parameters of Norway spruce (*Picea abies*) sample trees: number of resin canals (no. RC, cm^{-2}), total area of resin canals cm^{-2}, and number of epithelial cells (no. EC, cm^{-2}). The bark anatomical parameters do not differ significantly between treatments on any sampling date.

	22 May 2013			21 August 2013		
	C	SC	FC	C	SC	FC
n	4	5	6	6	6	6
no. RC cm^{-2}	66.65±10.32	54.38±5.10	52.52±5.96	54.82±3.85	42.74±2.01	50.70±4.49
total area RC cm^{-2}	79587.9±20809.2	67074.5±12084.1	61036.4±9658.4	50934.9±5774.3	46328.7±7962.0	54447.6±7336.7
no. EC cm^{-2}	470.50±123.08	405.18±44.82	421.76±59.26	399.66±24.53	313.89±25.73	381.97±40.77
